# False-Positive Human Immunodeficiency Virus Enzyme Immunoassay Results in Pregnant Women

**DOI:** 10.1371/journal.pone.0016538

**Published:** 2011-01-27

**Authors:** Laura G. Wesolowski, Kevin P. Delaney, Margaret A. Lampe, Steven R. Nesheim

**Affiliations:** Division of HIV/AIDS Prevention, National Center for HIV/AIDS, Viral Hepatitis, STD, and TB Prevention, Centers for Disease Control and Prevention, Atlanta, Georgia, United States of America; University of Rochester, United States of America

## Abstract

**Objective:**

Examine whether false-positive HIV enzyme immunoassay (EIA) test results occur more frequently among pregnant women than among women who are not pregnant and men (others).

**Design:**

To obtain a large number of pregnant women and others tested for HIV, we identified specimens tested at a national laboratory using Genetic Systems HIV-1/HIV-2 Plus O EIA from July 2007 to June 2008.

**Methods:**

Specimens with EIA repeatedly reactive and Western blot-negative or indeterminate results were considered EIA false-positive. We compared the false-positive rate among uninfected pregnant women and others, adjusting for HIV prevalence. Among all reactive EIAs, we evaluated the proportion of false-positives, positive predictive value (PPV), and Western blot bands among indeterminates, by pregnancy status.

**Results:**

HIV prevalence was 0.06% among 921,438 pregnant women and 1.34% among 1,103,961 others. The false-positive rate was lower for pregnant women than others (0.14% vs. 0.21%, odds ratio 0.65 [95% confidence interval 0.61, 0.70]). Pregnant women with reactive EIAs were more likely than others (p<0.01) to have Western blot-negative (52.9% vs. 9.8%) and indeterminate results (17.0% vs. 3.7%) and lower PPV (30% vs. 87%). The p24 band was detected more often among pregnant women (p<0.01).

**Conclusions:**

False-positive HIV EIA results were rare and occurred less frequently among pregnant women than others. Pregnant women with reactive EIAs were more likely to have negative and indeterminate Western blot results due to lower HIV prevalence and higher p24 reactivity, respectively. Indeterminate results may complicate clinical management during pregnancy. Alternative methods are needed to rule out infection in persons with reactive EIAs from low prevalence populations.

## Introduction

Universal screening to identify HIV infection in pregnant women is recommended so that infected women can be linked to care, start prophylaxis, plan for delivery, and avoid transmission through breastfeeding [1]–[4]. Screening is often conducted using a laboratory-based testing algorithm that incorporates enzyme immunoassays (EIAs) which have been approved by the Food and Drug Administration (FDA). These EIAs are highly sensitive and specific, but there is a perception that pregnant women are at higher risk for false-positive results [5], [6]. If this perception is shared by clinicians, they may be less likely to adopt universal screening. False-positive HIV screening test results occur when a repeatedly reactive EIA is followed by a negative or indeterminate confirmatory test result in someone who is not infected. A person whose specimen exhibits a repeatedly reactive EIA and negative confirmatory test is likely not infected, and follow-up testing should be based on risk behaviors [7]. Persons with an indeterminate Western blot who are at low risk for HIV infection, including most pregnant women in the United States, are often uninfected [8]. Persons with indeterminate results should be re-tested to resolve infection status a month after the initial Western blot, and if possible, pregnant women need to resolve their infection status before entering labor to plan for delivery if infected [3], [7]. False-positive HIV antibody test results can occur in the absence of infection due to cross-reactivity between viral proteins and tested specimens, but such cross-reactivity is less common using current peptide-based EIAs which contain fewer antigens than previous viral lysate-based EIAs [9].

Although a previous study indicated that parity is associated with false-positive HIV test results [10], it is not clear whether being pregnant at the time of an HIV test is associated. One study did not find pregnancy to be associated with indeterminate Western blot results in uninfected persons, but its power to detect an association was low [10]. Recent studies have evaluated EIA test performance among women in labor [11], [12]. These studies did not examine test specificity, which is inversely related to the proportion of false-positive results, among persons who were not pregnant. However, the confidence intervals for specificity for all EIA tests used on pregnant women, including rapid tests, overlapped the specificity figures listed in the FDA-cleared package inserts, which presumably used a non-pregnant population [11], [12] to determine assay performance. These studies suggest that the false-positive rate in pregnant women may not differ from that in non-pregnant persons, but they were not designed to make that comparison. Understanding the rate of false-positive EIA results in pregnant women is also necessary to gauge whether alternative algorithms, such as dual EIA algorithms, could be used in this population [13]. In order to evaluate the occurrence of false-positive HIV antibody test results in pregnant women compared with others tested for HIV, we retrospectively evaluated over three million HIV test results from laboratories operated by a large U.S. commercial laboratory, which is believed to be the largest such examination conducted to date.

## Methods

We retrospectively collected testing data without personal identifiers from serum and plasma specimens from persons 12 years of age and older that had been tested using the peptide-based Genetic Systems HIV-1/HIV-2 Plus O EIA (Bio-Rad, Redmond, Washington) at laboratories operated by a national laboratory from July 1, 2007, through June 30, 2008. Specimens with repeatedly reactive EIA results had been tested using the Genetic Systems HIV-1 Western blot kit (Bio-Rad, Redmond, Washington). EIA and Western blot tests were conducted according to manufacturer instructions. Specimens were categorized by pregnancy status based on whether they were obtained from persons who were pregnant, not pregnant, or persons for whom pregnancy status was unknown. A specimen was considered to be from a woman who was pregnant on the day the blood was collected for GS EIA testing if at least one of the following criteria was met: i) positive urine or serum human chorionic gonadotropin (HCG)-based pregnancy test, ii) a simultaneous request for either a cytogenetic test, a maternal serum screen panel, rubella serology as part of an obstetric panel, a one-hour glucose tolerance test for gestational diabetes, or iii) provision of an ICD-9 code for normal pregnancy or high-risk pregnancy or other pregnancy-related ICD-9 code. A specimen was categorized as being from a person who was non-pregnant when it was from: i) a male, ii) a female with a negative pregnancy test or ICD-9 code for a negative pregnancy test; iii) a woman age 55 or over, or iv) a female with an unspecified age who did not meet the above described pregnancy criteria. Specimens were categorized as being from a person whose pregnancy status was unknown when either: i) a female did not meet any of the pregnant or non-pregnant criteria listed above, ii) the submitted test requisition specified the individual as a male, but they simultaneously met one of the pregnancy-related criteria, or iii) gender was not specified. Criteria for these categories were based on discussions with staff from the American College of Obstetrics and Gynecologists coding department. Further, in a prospective study conducted at the same laboratories, all specimens from persons categorized as pregnant using these criteria (n = 474) were found to be pregnant on a quantitative HCG pregnancy test and 1430/1431 (99.93%) labeled as not pregnant using these criteria were found not to be pregnant using a quantitative HCG pregnancy test.

The number and percent of specimens with HIV test results in each of the following HIV infection categories were quantified. Specimens with HIV-negative EIA results were considered uninfected. Specimens with a repeatedly reactive EIA and positive Western blot were considered HIV-infected. A false-positive HIV test result was defined as a repeatedly reactive EIA followed by a negative or indeterminate Western blot result. The false-positive rate was defined as [# false-positive/# uninfected persons] where uninfected persons were considered those who were EIA-negative and those with false-positive results. The false-positive rate is equivalent to [1-specificity]. The false-positive rate was compared for: i) pregnant women versus persons who were non-pregnant, ii) for pregnant women versus women of reproductive age (12 to 55 years) who were non-pregnant, and iii) for pregnant women versus persons whose pregnancy status was unknown. We also examined the false-positive rate by the following co-factors: age, month of testing, and laboratory facility. Race/ethnicity data were not available. We analyzed the risk of false-positive HIV test result for pregnant women compared to persons who were not pregnant using a Mantel Haenszel odds ratio (OR) which was adjusted for HIV prevalence at each laboratory facility. The Centers for Disease Control and Prevention (CDC) received de-identified study data in aggregate, so multivariable regression techniques to adjust for co-factors beyond HIV prevalence at laboratories, which were related to pregnancy and false-positive HIV EIA test results, could not be performed.

Among all specimens with repeatedly reactive EIAs, we evaluated the proportion of specimens that were Western blot-negative, indeterminate, or positive, and the positive predictive value of the EIA test, by pregnancy status. Among those with Western blot-indeterminate results, we evaluated whether antibody reactivity to specific HIV polypeptide bands was detected more frequently among pregnant women than others. Statistical comparisons were made using the chi-square test to assess the difference between two proportions. The statistical software package SAS v9.1 (Cary, NC) was used for data analysis.

Since the laboratory dataset was based on distinct patient encounters, individuals may have been included in the analysis dataset more than once (i.e., if they had multiple blood collection events). It is unlikely that persons with EIA negative results had more than one test result during the analysis period, with the possible exception of pregnant women from areas of high HIV prevalence [2]. We assessed whether persons with a false-positive result had follow-up HIV testing by July 2008, by pregnancy status. A person was identified as having a follow-up specimen if name, date of birth and gender were the same as for the initial specimen because no additional patient identifiers were available. Among persons with repeatedly reactive EIAs and negative or indeterminate Western blot results, we assessed whether the follow-up test result was Western blot-positive within a month after the false-positive result. The one-month period was chosen to assess whether persons designated as having false-positive results appeared to have been infected at the time of that initial test, because most persons with indeterminate results who are infected with HIV-1 will develop detectable antibody within that one month follow up period [7], [14]. It is not known whether persons with false-positive HIV- 1/2 antibody EIA results had RNA or DNA testing to resolve infection status.

Probabilistic sensitivity analyses with 10,000 repetitions were conducted to evaluate the false-positive rate among pregnant women assuming that the proportion of specimens from persons of unknown pregnancy status to be categorized as pregnant was binomially distributed [15]. This proportion was estimated to be 23% based on data from a prospective study at the same commercial laboratory (data not shown). Probabilistic sensitivity analyses with 10,000 repetitions were also conducted to evaluate the false-positive rate among non-pregnant persons after (1) re-categorizing persons of unknown pregnancy status as non-pregnant as described above and (2) removing specimens with EIA false-positive results that were potentially infected. The proportion of potentially infected non-pregnant persons with repeatedly-reactive EIA and Western blot-negative specimens removed from analysis was sampled from a triangular distribution with a mode equal to the percent of repeatedly reactive EIA and Western blot-negative specimens with follow-up results that were Western blot-positive (18%), and with range based on plausible values from the literature (0.05% to 25%) [16]. Likewise, the proportion of potentially infected non-pregnant persons with repeatedly reactive EIA and indeterminate Western blot results removed from analysis was also selected from a triangular distribution with mode equal to the proportion with follow-up results that were Western blot-positive (35%), and with range based on plausible values from the literature (0.05% to 40%) [16]–[18]. In order to examine how the false-positive rate among non-pregnants would be impacted if a much greater proportion of those designated as false-positive were actually infected than that observed among those with follow-up HIV testing, we assessed the false-positive rate after doubling the mode values (i.e., mode = 36% in the EIA-repeatedly reactive and Western blot-negative group and mode = 70% in the Western blot-indeterminate group).

This study was determined to be research not involving identifiable human subjects by the National Center for HIV, Viral Hepatitis, STD, and TB Prevention at the Centers for Disease Control and Prevention. According to HIPPA regulations, protected health information can be disclosed, without the written authorization of the individual, to a public health entity for public health activities and purposes, such as this investigation. For this project, a laboratory shared health information (test results), without personal identifiers, with CDC.

## Results

During the analysis period, 3,357,200 specimens had an EIA test result. Of those, 921,501 (27.5%) were from pregnant women, 1,104,118 (32.9%) were from persons who were non-pregnant, and 1,331,581 (39.7%) were from persons whose pregnancy status was unknown (Table 1). The criteria identified most frequently for those categorized as pregnant was an ICD-9 code for normal or high-risk pregnancy (80.3%) and for those categorized as not pregnant was being male (87.4%) (Table 1).

**Table 1:** Pregnancy status of persons with specimens tested at a national commercial laboratory using Genetic Systems HIV-1/HIV-2 Plus O EIA (n = 3,357,200); July 2007 to June 2008.

Pregnancy Status	Pregnancy Status Criteria^a^	N (%)
Pregnant		
	Positive HCG test	31,897 (3.5)
	Cytogenetic tests	5 (0)
	Maternal serum screens	1,796 (0.2)
	ICD-9 codes for pregnancy	739,869 (80.3)
	Rubella test on obstetric panel	72,155 (7.8)
	One-hour glucose challenge test	3,101 (0.3)
	Other ICD-9 codes	72,678 (7.9)
	Total pregnant	921,501
Not pregnant		
	Males	964,592 (87.4)
	Females with negative HCG test	50,559 (4.6)
	Females with ICD-9 code 72.41^b^	192 (0)
	Females age 55 and over	88,416 (8.0)
	Females age unknown	359 (0)
	Total not pregnant	1,104,118 (32.9)
Pregnancy Unknown		
	Females unknown pregnancy status	1,292,938 (97.1)
	Gender not specified	36,133 (2.7)
	Coded as males and pregnant	2,510 (0.2)
	Total Pregnancy Unknown	1,331,581 (39.7)

a Criteria used to establish pregnancy status are listed in the order they were evaluated.

b Negative pregnancy test result.

Of 3,356,764 (99.9%) specimens with interpretable HIV test results, 541 (0.06%) of 921,438 from pregnant women and 14,788 (1.34%) of 1,103,961 from persons who were non-pregnant were Western blot-positive (Table 2). The false-positive HIV EIA rate for pregnant women was lower than for persons who were non-pregnant (0.14% vs. 0.21%, p<0.01), and it was lower for pregnant women than for persons of unknown pregnancy status (0.14% vs. 0.18%, p<0.01) (Table 2). The false-positive HIV EIA rate for pregnant women (0.14%) was not different than that for women of reproductive age who were non-pregnant [0.15%, (74/50,565), p = 0.56].

**Table 2:** HIV test results, false-positive rate and risk of false-positive result, by pregnancy status^a^, national commercial laboratory, July 2007 to June 2008.

	EIAnon-reactiveN (%)	Repeatedly-reactive EIAWestern blot- positiveN (%)	Repeatedly reactive EIAWestern blot- negativeN(%)	Repeatedly reactive EIAWestern blot- indeterminateN(%)	False positive rate^b^	Crude odds ratio (95% CI)	Adjustedodds ratio^c^(95% CI)
Pregnant	919,640 (99.8)	541(0.06)	951(0.10)	306(0.03)	0.14%	0.65(0.60, 0.69)	0.65(0.61, 0.70)
Not Pregnant	1,086,865 (98.5)	14,788 (1.34)	1,675(0.15)	633(0.06)	0.21%	Reference	Reference
Pregnancy Unknown	1,324,344 (99.5)	4,617(0.35)	1,703(0.13)	701(0.05)	0.18%	0.86(0.81, 0.91)	0.85(0.80, 0.90)

a Excludes 436 with uninterpretable Western blots or repeatedly-reactive EIA with Western blot not performed.

b False positive = EIA repeatedly-reactive and Western blot negative or indeterminate

False positive rate =  [false-positive/(EIA-non-reactive + false-positive)].

c Adjusted for laboratory HIV prevalence.

The false-positive rate was lower in those study subjects who were the median age of 30.2 years or younger [0.17% (2,789/1,646,060)] compared with those who were older than 30.2 [0.19% (3,056/1,651,993), p<0.01]. The median monthly HIV EIA false-positive rate for this one-year study period was 0.17%, and ranged from 0.16% to 0.21%. The occurrence of false-positives did not appear to be seasonal (not shown). The median false-positive rate by individual laboratory facility was 0.14% (range 0.04% to 0.65%). The two laboratory facilities with the highest HIV EIA false-positive rates function primarily as reference laboratories for hospitals and other facilities, and are more likely to receive specimens that initially screened HIV-repeatedly reactive than those tested at other regional facilities within the same laboratory system. The HIV prevalence at the laboratory facilities ranged from 0.17% to 2.8%, and the two reference laboratories mentioned previously had the highest prevalence rates. After adjusting for prevalence at each laboratory facility, pregnant women were less likely to have false-positive screening test results than non-pregnant persons [adjusted OR 0.65, 95% confidence interval (CI) (0.61, 0.70)] (Table 2).

Among all specimens with repeatedly reactive HIV EIA results, those from pregnant women were more likely to test Western blot-negative and indeterminate than those from persons who were not pregnant (52.9% vs. 9.8%, p<0.01) and (17.0% vs. 3.7%, p<0.01), respectively (Table 3). Among persons with indeterminate Western blot results, the only band detected more often among pregnant women than among persons who were not pregnant was the p24 band (79% vs. 68%, p<0.01). The positive predictive value of the HIV EIA test among pregnant women was lower than that among persons who were not pregnant (30% vs. 86.5%, p<0.01) (Table 3).

**Table 3:** Among specimens with a repeatedly-reactive EIA, Western blot result and positive predictive value, by pregnancy status^a^; national commercial laboratory, July 2007 to June 2008.

	PregnantN(%)	Not PregnantN(%)	p-value
Western blot result			
Negative	951 (52.9)	1,675 (9.8)	p<0.01
Indeterminate	306 (17.0)	633 (3.7)	p<0.01
Positive	541 (30.0)^b^	14,788 (86.5)^b^	p<0.01
Total	1,798	17,096	

a Excludes 436 with uninterpretable Western blots or repeatedly-reactive EIAs with Western blot not performed.

b Positive predictive value.

Of 4,329 specimens with repeatedly reactive EIA and Western blot-negative results, 346 (8.0%) had at least one follow-up testing event by July 2008: 106/951 (11.2%) pregnant females, 119/1675 (7.1%) non-pregnant persons, and 121/1703 (7.1%) persons with unknown pregnancy status. Of those with follow-up test results within a month following the initial EIA-reactive and Western blot-negative result, fewer pregnant women than non-pregnant persons had a Western blot-positive result [0/54 (0%) vs. 12/56 (21.4%), p<0.01]. Nine non-pregnant persons and no pregnant women had follow-up results between 31 days and one year that were Western blot-positive.

Of 1,640 specimens with repeatedly-reactive HIV EIA and Western blot-indeterminate results, 187 (11.4%) had at least one follow-up testing event by July 2008: 70/306 (22.9%) pregnant women, 57/633 (9.0%) non-pregnant, and 60/701 (8.6%) unknown pregnancy status. Slightly more than half (57.2%) of the persons with follow-up test results within a month after the initial indeterminate Western blot result had follow-up testing. Fewer pregnant women than non-pregnant persons had a positive Western blot result within one month of their indeterminate result [(0/39 (0%) vs. 13/34 (38.2%), p<0.01]. Two pregnant women and seven non-pregnant persons with initial results that were indeterminate had follow-up results between 31 days and one year that were Western blot-positive.

Following the sensitivity analysis in which specimens were re-categorized as pregnant from the pregnancy-unknown category, the false-positive rate among pregnant persons was 0.15%. After specimens were categorized as non-pregnant from the pregnancy-unknown category and approximately 18% (21/119) of repeatedly reactive EIA and Western blot-negative and 35% (20/57) of indeterminate specimens were reclassified because they may have represented true infections, the false-positive rate among non-pregnant persons was 0.16%. If the proportion misclassified as false-positive were twice the rate observed among those designated as false-positive with follow-up testing, the false-positive rate among non-pregnant persons would be higher than 0.15%, the false-positive EIA rate among pregnant women based on this sensitivity analysis.

## Discussion

We examined over three million HIV EIA test results and found that false-positive results were rare (less than two in a thousand) and occurred at a rate similar to that described in the manufacturer's package insert (Bio-Rad, Redmond, Washington). Further, they occurred less frequently among persons who were pregnant (0.14%) than among persons who were not pregnant (0.21%). It is possible that the false-positive rate was higher in non-pregnant persons because some were actually infected, and in the process of seroconversion, particularly non-pregnant persons with indeterminate results showing viral bands who reside in areas of high prevalence and have other risk factors for HIV. Ideally, truly HIV-infected persons would have positive confirmatory results instead of indeterminate Western blot results, but new EIAs can detect infections earlier than the Western blot develops the bands needed to be considered positive [19]. Nevertheless, when the proportion of specimens found to be infected on follow-up were removed, pregnant women were not more likely to have false-positive HIV EIA test results than others testing for HIV, as previously thought. Basing the proportion of false-positives among non-pregnants misclassified as infected on those with follow-up testing is likely to artificially reduce the number classified as false-positive because those with follow-up are more likely to be infected than those without it. The observed difference in the false-positive rate by pregnancy status, which amounts to less than one false-positive result per one thousand tests, could also be explained by observed differences in the false-positive rate by laboratory or other unmeasured characteristics such as a concurrent medical condition.

In this study and others, repeatedly reactive HIV EIA results were unlikely to be indicative of HIV infection in pregnant women [12]. This is expected because as disease prevalence decreases, the proportion of reactive tests which are false positive increases because there are fewer infected persons in the population being tested. In this study, pregnant women with indeterminate Western blot results were often uninfected, based on data from a limited number of women with follow-up tests and the low prevalence of HIV infection in this population. Indeterminate Western blots among pregnant women frequently displayed reactivity with the p24 band, a band often observed in individuals with low risk of infection (8). Recommendations for testing following an indeterminate Western blot include conducting a Western blot or indirect immunofluorescence assay on a second sample at least one month after the indeterminate result, or testing for the presence of HIV nucleic acids [7]. Obtaining testing several weeks after an initial indeterminate Western blot result may not be practical for pregnant women, as evidenced by the very low proportion in our study (23%) with follow-up Western blot testing. Some practitioners recommend using a DNA polymerase chain reaction method to test for infection in a pregnant woman with indeterminate results, but these tests are not currently approved by the FDA for diagnostic purposes, may not be available, are expensive, and require skilled technicians (5). A person with a reactive EIA and Western blot negative or indeterminate result may have an approved nucleic acid amplification test, and if positive, can be considered infected, but further serologic testing is required for a person with a reactive EIA and negative nucleic acid test results (APTIMA HIV-1 RNA Qualitative Assay, GEN-PROBE, San Diego, CA). Also, although nucleic acid testing is sensitive for detecting early infection, it is less sensitive for the detection of established infection than serologic tests [19], [20].

Pregnant women with reactive HIV EIA results would benefit from a testing algorithm which would accurately rule-out HIV infection status in a timely way before the onset of labor, without the provision of indeterminate results which may cause undue stress in someone who is not infected. It is not yet known whether an algorithm using two antibody EIAs with different antigen or binding properties in series could be used in pregnant women with a reactive screening result or whether both EIAs would have concurrent non-specific reactivity in uninfected persons [13]. It is ideal to establish HIV infection status before a pregnant woman goes into labor, but if infection status is unknown at the time of labor, a rapid test is conducted, and if positive, antiretroviral therapy is recommended [21].

This study was subject to several limitations. Study participants could have been included in the study more than one time, but it is unlikely that de-duplicating would impact the findings, as few subjects repeated testing even when this testing was recommended. Persons with negative EIA results are unlikely to have had follow-up testing, and slight changes to the large number of non-reactive tests would have little impact on false-positive rates. Pregnant women with false-positive HIV EIA screening results were more likely to have recommended repeat testing, so if these follow-up specimens were de-duplicated, the number of false-positives among pregnant women would decrease and the rate would still be lower than that among non-pregnant persons. The rate of false-positive EIAs in pregnant women and others may vary by the EIA and supplemental test used, although since the prevalence of HIV in pregnant women in the US tends to be low, the predictive value of a positive EIA screening test result is likely to remain low regardless of the screening test used. There is a possibility of misclassification of pregnancy status because few persons classified as pregnant had concomitant pregnancy tests, however, most persons were categorized as pregnant based on an ICD-9 code for pregnancy, so misclassification is unlikely to be extensive, and HCG tests conducted on prospectively collected specimens indicate that this misclassification was likely very limited. Precise estimates of the proportion of pregnant women and others with repeatedly reactive EIA results and negative or indeterminate Western blot results which are truly infected based on nucleic acid testing or other follow-up testing are not available. The precision of the sensitivity analyses reported here could be improved with better estimates of the rate of such misclassification. Ideally, additional variables such as age and race could have been included in a multivariable model examining false-positive results by pregnancy status. The false positive rate was statistically lower in persons younger than the median age, but the difference was 2 in 10,000, which may not be meaningfully different, so it may not be a strong confounder. Finally, 359 women with an unspecified age should have been included in the pregnancy unknown category instead of the not pregnant category, but they could not be re-categorized because we did not receive line level data. However, they constituted approximately 0.03% of the not pregnant group, so the impact of re-categorizing their pregnancy status on study findings would be negligible.

Approximately 70% of pregnant women in the United States receive prenatal antibody screening for HIV infection, and increasing this proportion is necessary given that approximately one quarter of new HIV infections occur among women, many of whom are of child-bearing age [22], [23]. False-positive antibody EIA test results are rare, so universal HIV screening among pregnant women should be pursued without hesitation unless a woman declines [2]. However, clinicians should be aware that when HIV prevalence is low, as is often the case among pregnant women in the United States, a reactive EIA result is more likely to be false-positive. Testing strategies that allow for more timely and accurate identification of false-positive HIV antibody test results should be considered for low prevalence populations, including pregnant women [13].
